# Monthly Summary

**Published:** 1870-01

**Authors:** 


### MONTHLY SUMMARY.

---

*A Valuable Cement.*—Glycerine and litharge, mixed into a paste, furnish an extremely firm cement for iron and stone, as well as fastening iron to iron, and is said to be particularly adapted to fixing iron to stone, as for railways, etc. The material hardens very quickly, and must, therefore, be used at once. It is insoluble in water, and only attacked by concentrated acids. Articles joined with it can be used in a very few hours afterwards. Sandstone blocks, joined by this cement, have broken in a fresh fracture, rather than at the point of the union of the original surfaces. Very dry litharge does not form so good a cement as that which has absorbed a considerable amount of water. Only the purest material is to be used.—*Med. and Surg. Reporter.*

---

*Large Salivary Calculi.*—Dr. H. E. Von Rygersma has removed several very large salivary calculi from the mouth of a negro man about 50 years old. The first was extracted by a French surgeon, and was the size of a pigeon's egg. Two were as large as Lima beans. The excretory duct of the submaxillary gland is described as completely changed into a bag, and will readily admit a grain of corn.—*Med. and Surg. Reporter.*

---

*Soothing Syrup.*—A correspondont of the *California Medical Gazette* publishes an analysis of "Mrs. Winslow's Soothing Syrup" which will astonish even those who are aware of the deleterious properties of that nostrum. Ten drachms of the syrup yielded 1.14 grain of morphine and other opium alkaloids, "very nearly one grain to the ounce of syrup." If used according to the printed directions, "we have a dose of morphine equal to ten drops of laudanum, given to a child of three months old every two hours, and double the quantity to a child of six months old." Is infant mortality to be wondered at when mothers and nurses deal in such deadly compounds?—*Med. Gazette.*

---

*Death from a Dissecting Wound.*—Our exchanges inform us that Prof. Bœhm, one of the most eminent medical men of Berlin, died a few days since under fearful circumstances ; while dissecting before a class of students he pricked a finger. He thought it a mere abrasion of the skin and failed to cauterize it. Two days afterwards his hand began to swell, and became enormous. The poison pervaded his whole system and killed him. He retained his conciousness nearly to the last, and saw his end approach with undisturbed firmness.

*Painless Cutting in Surgery.*—The *Lancet* informs us that "at at late meeting of the British Medical Association, Dr. B. W. Richardson exhibited a knife consisting of a revolving blade, and which divided with such rapidity that superficial incisions could be made with it without pain. The revolutions were about twenty-five per second, but the speed might be greatly increased. The knife in its action illustrated that an appreciable interval of time is necessary for fixing an impression on the mind, and for the development of consciousness. He hoped he should soon be able to give to the surgeon a small pocket instrument with which to open abscesses, and perform many minor surgical operations painlessly, without having recourse to either general or local anæsthesia."

---

*An Ingenious Method for Drying Vegetable and Animal Substances.*—A method recently adopted for drying vegetable and animal substances, consists in filling a vessel half full with fused chloride of calcium, pouring ether upon it, and then placing above it a vessel containing the material to be dried. The vessel is placed upon a glass plate, and over this a bell-glass fitting completely to its surface. The chloride of calcium abstracts the the moisture from the ether, which then constantly takes away a new quantity from the substance in the vessel above, until it is quite dry. Articles dried in this manner have quite a different appearance from those from which the moisture is removed by by the ordinary process, vegetables retaining their natural color, and animal substances their elasticity and flexibility.—*Med. and Surg. Reporter.*

---

*Warts.*—The modern scientific name of this disorder is *Myrmekiasmos*, but the principal point is the surprising rapidity with which an extensive cluster of warts will disappear under the use of arsenic, without any local application whatever.

Potassa fusa has been used locally. The warts should be saturated with a solution of equal parts of potassa fusa and water, until the horny growth is dissolved and the papillæ destroyed by this caustic.—*Med. Gazette.*

